# Chemistry: General Medical and Pharmaceutical

**Published:** 1883-11

**Authors:** 


**Chemistry: General Medical and Pharmaceutical.** Including the Chemistry of the U. S. Pharmacopœia. A manual of the general principles of the science and their application in medicine and pharmacy. By JOHN ATTFIELD, F. R. S., M. D., Ph. D., Professor of Practical Chemistry to the Pharmaceutical Society of Great Britain. Fifth edition, specially revised by the author for America. Philadelphia: H. C. Lea's Son & Co. 1883.

This book has met with so signal a success as to place it beyond criticism. Within sixteen years the demand has called for ten large editions of this manual. In our opinion it is by far the best work for the use of medical students, as the laws and principles of the science of chemistry are elucidated and illustrated by that portion of chemistry in which the medical practitioner is directly interested. In this respect it is in happy contrast with the many works now offered to the profession, in which the illustrations and experiments given apparently ignore the abundance of material which has this direct relationship.

